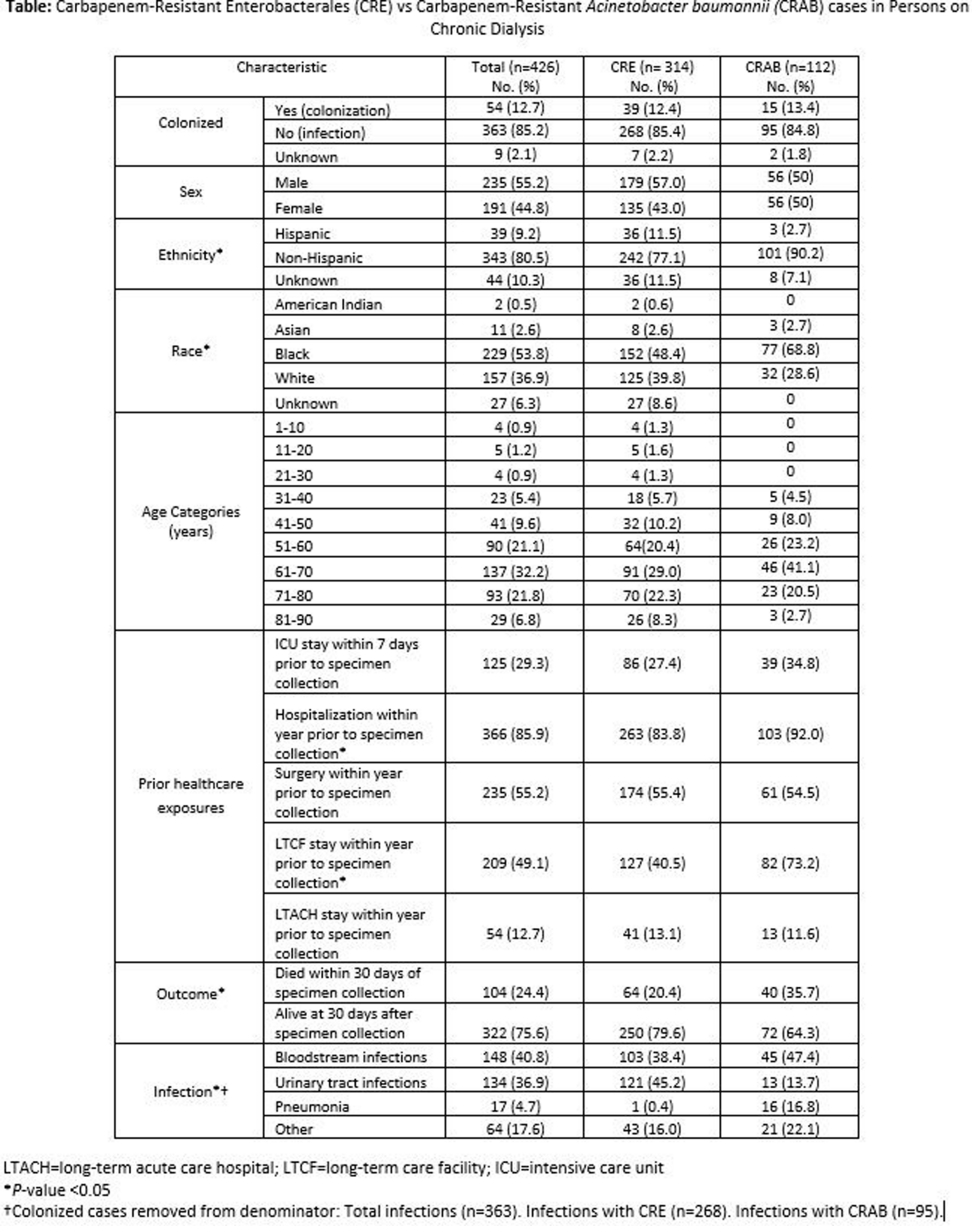# Carbapenem-resistant Acinetobacter baumannii and Carbapenem-resistant Enterobacterales in US Dialysis Populations, 2016-2021

**DOI:** 10.1017/ash.2024.225

**Published:** 2024-09-16

**Authors:** Danica Gomes, Julian Grass, Sandra Bulens, Nadezhda Duffy, Joshua Brandenburg, Jesse Jacob, Gillian Smith, Elisabeth Vaeth, Ghinwa Dumyati, Kristina Flores, Christopher Wilson, Daniel Muleta, Christopher Czaja, Helen Johnston, Ruth Lynfield, Paula Snippes Vagnone, Sean O’Malley, Nicole Stabach, Joelle Nadle, Rebecca Pierce, Alice Guh, Shannon Novosad, P. Maureen Cassidy

**Affiliations:** Centers for Disease Control and Prevention; Division of Healthcare Quality Promotion, Centers for Disease Control and Prevention; Emory University; Georgia Emerging Infections Program; Maryland Department of Health; University of Rochester Medical Center; University of New Mexico; Tennessee Department of Health; Colorado Department of Public Health and Environment; Minnesota Dept of Health; MN Dept. of Health, Public Health Lab; Conneticut Department of Public Health, Healthcare Associated Infections & Antimicrobial Resistance Program; California Emerging Infections Program; Oregon Health Authority; Oregon Public Health Division

## Abstract

**Background:** Infections lead to high mortality among patients on chronic dialysis; knowledge of multi-drug resistant infections is limited. The Centers for Disease Control and Prevention’s Emerging Infections Program (EIP) conducts laboratory- and population-based surveillance for carbapenem-resistant Enterobacterales (CRE) in 10 U.S. sites and carbapenem-resistant Acinetobacter baumannii (CRAB) in 9 U.S. sites. We investigated clinical characteristics, healthcare exposures, and outcomes of CRE and CRAB cases in persons on chronic dialysis from 2016-2021. **Methods:** Among EIP catchment-area residents on chronic dialysis, we defined a CRE case as the first isolation of Escherichia coli, Enterobacter cloacae complex, Klebsiella aerogenes (formerly Enterobacter aerogenes), Klebsiella oxytoca, Klebsiella pneumoniae, or Klebsiella variicola resistant to any carbapenem, from a normally sterile site or urine in a 30-day period. A CRAB case was defined as the first isolation of Acinetobacter baumannii complex resistant to any carbapenem (excluding ertapenem), from a normally sterile site or urine (or lower respiratory tract or wound since 2021) in a 30-day period. Medical records were reviewed. A case was considered colonized if the case culture had no associated infection type or colonization was documented in the medical record. Descriptive analyses, including analyses stratified by pathogen, were conducted. **Results:** Among 426 cases, 314 were CRE, and 112 were CRAB; most cases were male (235, 55.2%), Black (229, 53.8%), and 51-80 years old (320, 75.1%) (Table). An infection was associated with 363 (85.2%) case cultures; bloodstream infections (148; 40.8%), urinary tract infections (134; 36.9%), and pneumonia (17; 4.7%) were the most frequent. Overall, most cases had documented healthcare exposures (excluding outpatient dialysis) in the year before incident specimen collection, including: 366 (85.9%) hospitalizations, 235 (55.2%) surgeries, 209 (49.1%) long-term care facility stays, 54 (12.7%) long-term acute care facility stays. Additionally, 125 (29.3%) had an intensive care unit admission within the 7 days before incident specimen collection. Compared to CRE cases, a higher proportion of CRAB cases (a) had a long-term care facility stay (82/112 [73.2%] versus 127/314 [40.5%], P<.0001) or hospitalization (103/112 [92%] versus 263/314 [83.8%], P = .03) within the preceding year and (b) died within 30 days of incident specimen collection (40/112 [35.7%] versus 64/314 [20.4%], P = .001). **Discussion:** Among CRE and CRAB cases in persons on chronic dialysis, healthcare exposures were common, and mortality was high. Additional efforts to better describe the burden of these organisms and associated risk factors in the dialysis population are needed for tailoring infection prevention strategies to this vulnerable.

**Figure:**